# Text Message-Based Cessation Intervention for People Who Smoked or Used Smokeless Tobacco in India: A Feasibility Randomized Controlled Trial

**DOI:** 10.1093/ntr/ntae056

**Published:** 2024-03-12

**Authors:** Abhijit Nadkarni, Leena Gaikwad, Miriam Sequeira, Joseline D’souza, Megan Lopes, Rajanish Haldankar, Pratima Murthy, Richard Velleman, Urvita Bhatia, Felix Naughton

**Affiliations:** Centre for Global Mental Health, Department of Population Health, London School of Hygiene and Tropical Medicine, London, UK; Addictions and Related Research Group, Sangath, Goa, India; Addictions and Related Research Group, Sangath, Goa, India; Addictions and Related Research Group, Sangath, Goa, India; Addictions and Related Research Group, Sangath, Goa, India; Addictions and Related Research Group, Sangath, Goa, India; Addictions and Related Research Group, Sangath, Goa, India; National Institute of Mental Health and Neurosciences, Bengaluru, India; Addictions and Related Research Group, Sangath, Goa, India; Department of Psychology, University of Bath, Bath, UK; Addictions and Related Research Group, Sangath, Goa, India; Department of Psychology, Health and Professional Development, Oxford Brookes University, Oxford, UK; School of Health Sciences, University of East Anglia, Norwich, UK

## Abstract

**Introduction:**

Despite the high burden of tobacco use in India, users do not have access to adequate help. This pilot trial aimed to evaluate the feasibility and acceptability of a text messaging intervention for tobacco cessation, generate preliminary estimates of its impact, and fine-tune procedures for a definitive trial.

**Aims and Methods:**

Parallel two-arm single blind individually randomized controlled pilot trial with nested qualitative study. Participants included adult current tobacco users (smoked and smokeless). Eligible and consenting participants were randomized to receive either (1) text messaging intervention (ToQuit) which covered specific content areas such as psychoeducation about consequences of tobacco use and benefits of quitting and tobacco avoidance strategies or (2) information about tobacco cessation helplines such as the helpline number and the languages in which tobacco cessation support was available (control). Feasibility data included screening and consent rates, treatment dropouts, and outcome ascertainment. The primary abstinence outcome was self-reported abstinence from tobacco in the past seven days at 3 months post-randomization. In-depth interviews were conducted with a subsample of participants primarily to collect acceptability data. The primary abstinence analysis used a chi-squared test and logistic regression (complete case), and qualitative data was analyzed using thematic analysis.

**Results:**

Ninety-eight participants were randomized into the two trial arms; 77 (79%) completed outcome evaluation. No between-arm differences in abstinence were found though findings favored the intervention (7-day abstinence: ToQuit 23%, control 19%; adjusted odds ratio 1.23, 95% confidence interval 0.38, 3.97). Participants appreciated the language, comprehensibility, and relevance of the messages; and reported overall satisfaction with and positive impact from the intervention on their lives.

**Conclusions:**

The findings indicate the acceptability and feasibility of ToQuit and if found effective, it could be a potentially scalable first-line response to tobacco use in low-resource settings.

**Implications:**

Our pilot randomized control trial provides sufficient findings supporting the acceptability and feasibility of an intervention for tobacco cessation which is suitable for a context which has a shortage of healthcare workers and for individuals who use smoked or smokeless tobacco. This is critical on a background of limited contextually relevant interventions for a problem with a high burden in low- and middle-income countries such as India.

## Introduction

Tobacco use is associated with high mortality, and a range of morbidities including cancer, and lung and cardiovascular diseases.^1^ Furthermore, premature deaths due to tobacco use deprive families of income, raise the cost of health care, and hinder economic development.^2^ 40% of the total economic cost because of tobacco use occurs in low- and middle-income countries (LMICs), and 25% is accounted for by Brazil, Russia, India, and China alone.^3^

India is the second largest consumer (current use- 48% males, 20% females; 275 million adults),^4^ and third largest producer of tobacco in the world^5^; and has one of the highest mortality rates related to tobacco (~0.9 million of the 6 million annual deaths globally).^2,6^ The treatment gap (proportion of people with a disorder who require an intervention but do not receive one) for tobacco use in India is as high as 92%.^7^ A recent study has shown that only 27% of people who use smokeless tobacco and 46% of people who smoke in India received advice to quit from a healthcare provider.^4^ Opportunistic screening and brief interventions in primary care, although recommended by the WHO, are largely missing in current practice in India.^8^ A key barrier is the shortage of healthcare workers, and hence non-resource-intensive interventions are needed to help the target population in resource-constrained settings.^6^ The existing tobacco cessation strategy in India has resulted in limited success because of poor availability and accessibility of services, and non-availability of interventions that are both, culturally relevant and contextual to the target population.^6^

Our study aims to overcome these accessibility and feasibility hurdles by developing and evaluating a tobacco-cessation intervention that can be delivered using low-cost and easily available mobile text messaging. India has 1.2 billion mobile phone subscriptions, or 84 telephone connections per 100 people,^9^ all of which will have bundled text messaging services. This provides an unique opportunity to significantly increase the penetration and coverage of tobacco cessation interventions in a low health-resource setting. Although there is moderate certainty evidence that automated text message cessation interventions are effective, evidence from LMICs is sparse, none have been developed for people who use smokeless tobacco, and the one scaled up for use in India has not been tested for effectiveness.^10,11^ Through a systematic and participatory formative research process we have iteratively developed a text messaging intervention called “ToQuit.” The feasibility randomized control trial (RCT) described in this paper aimed to empirically evaluate the feasibility and acceptability of the intervention, and generate preliminary estimates of its impact.

## Materials and Methods

### Design

This was a parallel-arm individual randomized controlled trial, with a nested qualitative study. The trial was preregistered on clinicaltrials.gov (ID: NCT04782453). Participants were recruited from April 12, 2021 to June 18, 2021; and the final outcome assessment was done on October 21, 2021.

### Sample

We aimed to recruit a sample of 100 participants, 50 each in the intervention and control groups. Based on our previous experience of feasibility RCTs and guidance on sample size requirements,^12^ this sample size was deemed sufficient to achieve the goals of the study.

Individuals were eligible for inclusion if they were adults (≥18 years), were currently using either smoked or smokeless tobacco (ie, at least once in the past 28 days), were willing to quit tobacco in the next 2 months, had a personal mobile phone, and were able to read and reply to an SMS text in English or Hindi.

### Recruitment

As the coronavirus disease-2019 (COVID-19) pandemic precluded face-to-face recruitment as originally planned, all recruitment procedures were conducted virtually and hence participants residing in any part of India were potentially eligible. Recruitment advertisements were shared over the institutional social media accounts (Facebook, Instagram, and Twitter) as well as the social media networks of the researchers. Individuals willing to quit tobacco called our designated number, sent a ‘ToQuit’ SMS to the number, or messaged us on our social media accounts. When a potential participant contacted us, a researcher called them back to seek informed consent. After collecting baseline data from consenting participants we conducted stratified randomization using a randomization sequence generated by a data manager using www.sealedenvelopes.com. Participants were randomly allocated to ToQuit or control group, in 1:1 ratio, stratified based on type of tobacco (people who primarily used smokeless tobacco or those who primarily smoked), in block sizes of two and four, using Sequentially Numbered Opaque Sealed Envelopes to maximize allocation concealment.

### Intervention

Participants in the intervention arm received a total of 124 messages on their mobile phones for 3–4 days a week over 8 weeks with three to five messages a day. Messages were delivered on their preferred days and time slots (8 to 10 AM, 12 to 3 PM, and 6 to 9 PM). These time slots were based on our formative research in which people who used tobacco indicated that the messages should be sent when the chances of using tobacco were the greatest and this was usually after waking up in the morning, after lunch, after their workday ended. On the last day of the intervention, they were asked to reply back with numbers (1- yes, 2- no) indicating whether they had managed to quit, if they wanted additional resources and helpline numbers and if they would recommend this intervention to others. The intervention messages (Appendix S1) covered specific content areas such as psychoeducation about consequences of tobacco use and benefits of quitting; goal setting; managing goals and self-monitoring of behavior; tobacco avoidance strategies; self-awareness and reflection messages; how to seek social and pharmacological support; identifying and managing cravings; and relapse prevention strategies. Some examples of messages within these content areas are as follows: Psychoeducation (“Every year 10 lakh people die in India because of tobacco use- most by heart attacks or cancer. Choose to quit and avoid being 1 of these 10 lakhs.”), goal setting (“People who can imagine themselves quitting tobacco are more likely to be successful in doing so. Imagine a fitter, stronger and healthier you and set a date to quit tobacco entirely.”), and identifying triggers and avoidance (“Remove all tobacco-related things from your home. It is easier when you do not see or smell tobacco. You can ask others not to use in front of you too.”).

Participants from the control group were given details of functional tobacco cessation helplines in India. This was in the form of a digital leaflet which was sent to them if they were randomized to the control arm. The leaflet contained details such as the name of the helpline, the helpline number, and the languages covered.

### Baseline Assessment

[1] Sociodemographic data such as age, gender, employment status, educational status, and marital status.
[2] Tobacco use including use of smoked and smokeless tobacco.
[3] Six questions about tobacco use from the Alcohol, Smoking, and Substance Involvement Screening Test (ASSIST) a tool developed by WHO to screen for substance use disorders related to alcohol, tobacco, and illicit drugs. ASSIST has good reliability and feasibility in culturally diverse settings and with different substance use patterns.^13^ It covers the following domains over the past 3 months: Frequency of use, urge to use, consequences (health, social, legal, or financial problems) of use, failure to do what is normally expected because of use, expressed concern about use by others, and failure to control, cut down or stop using.

### Outcome Assessment

Outcome data were collected at 3 months post-randomization regardless of treatment completion. The primary outcome was self-reported abstinence from tobacco in the past seven days. Secondary outcomes included mean change in ASSIST tobacco use sub-scale score and self-reported abstinence from tobacco in the past 28 days. The PI and outcome assessors were masked to the allocation of participants. In some cases where the participants consented to the qualitative interviews to be conducted immediately after the outcome evaluation, the outcome assessors were unblinded after asking questions about the primary and secondary outcomes.

### Feasibility Outcomes

We collected the following data during the course of the study: Number and proportion of participants consenting to the intervention, number and proportion of treatment dropouts (defined as participants choosing to stop receiving the intervention messages), reasons for refusal to participate, reasons for dropout, and number and proportion of participants completing outcome assessments.

### Nested Qualitative Study

Qualitative in-depth interviews (IDIs) were conducted with a subsample of participants from the intervention group purposively selecting those who reported abstinence and those who continued to use tobacco. We invited all intervention arm participants who completed outcome evaluation to participate in the qualitative study. Those who consented were sequentially recruited and we stopped recruitment after we reached data saturation. The IDIs were conducted after the 3-month outcome evaluation, in English or the vernacular language as appropriate. The IDIs were conducted over the phone by trained and experienced research assistants. The interviews were audio recorded, transcribed and the vernacular transcripts were translated into English before analysis.

We used a semi-structured interview guide (Appendix S2) that focused on understanding the acceptability, feasibility, and perceived impact of the intervention. The domains covered in the interview guide include (1) overall experience (eg, how was your overall experience with this intervention through mobile messaging?), (2) perceived impact (eg, during this 2-month intervention, were there changes in your tobacco use? Why do you think this change occurred?), (3) content (eg, is there any particular type of message that you found more helpful or less helpful? What are your thoughts on the language or wording of the messages?), (4) delivery (eg, what do you think about the length of the messages you received? What are your views on the frequency of the messages? What are your views on the time or the days on which you received the messages?), and (5) situational factors (eg, tell me about any circumstances, such as activities or work that you were involved in or any other factors that helped your participation in this intervention.).

Serious adverse events were monitored during the entire duration of the trial, and reported and managed as per the institution’s ethics committee guidelines.

### Analysis

Quantitative outcome data were analyzed using STATA 17, and reported consistent with CONSORT guidelines for parallel-arm randomized controlled trials.^14^ Baseline characteristics of participants were compared between those who completed outcome assessments and those who did not, and those in the intervention and control arms. Proportions or means were compared as appropriate using chi-square test and *t*-test respectively. The primary abstinence analysis was complete case analysis, after adjusting for baseline variables with imbalance, if any. The secondary analysis was intention-to-treat; missing outcome data were imputed using multiple imputation. Employment status and age category were used in the imputation model as there was imbalance between the two arms on these variables based on visual inspection. Regression analysis was conducted and effect size was reported as odds ratio with 95% confidence interval for categorical outcomes (point prevalence abstinence), and standardized mean differences with 95% confidence intervals for continuous outcomes (change in ASSIST scores between baseline and follow-up). The ‘mi estimate’ command was used to run the analytic model of interest (eg, logistic regression) within each of the imputed datasets as it also combines all the estimates across all the imputed datasets. Sensitivity analysis was conducted assuming data missing at random, and outcome groups were compared by replacing missing values with “abstinence achieved” as “best case scenario” and by failure to achieve the abstinence as “worst case scenario” for binary outcomes and with mean change in ASSIST score for the continuous outcome.

Thematic analysis was used to analyze the qualitative data, using N-vivo version 12. It consisted of an iterative process where a priori themes that mirrored the study goals about feasibility, and reflected in the domains covered by the intervention guide (eg, intervention content), were applied to the data. First, the researchers familiarized themselves with the data by reading interview transcripts. Subsequent analysis involved the generation of codes from raw data. These codes were used to inductively generate a new coding template, which was then applied to the remaining interviews. The first five transcripts were coded by one researcher and checked by the supervisor to develop the codebook. The remaining transcripts were single-coded independently by two coders. Themes were derived by retrieving pieces of data pertaining to codes and by examining their meaning in relation to the research questions. Patterns were derived by eliciting similarities and differences between participants on these themes. All the coders had direct experience with the study setting. The perspectives of the wider research team on this project facilitated the interpretation of the findings as related to a priori theoretical and empirical foundations of complex intervention testing in the study settings.

### Ethics

Ethical approval for this study has been obtained from the implementing organization’s Institutional Review Board and the ethics committee of the Indian Council of Medical Research.

## Results

**Figure 1.**
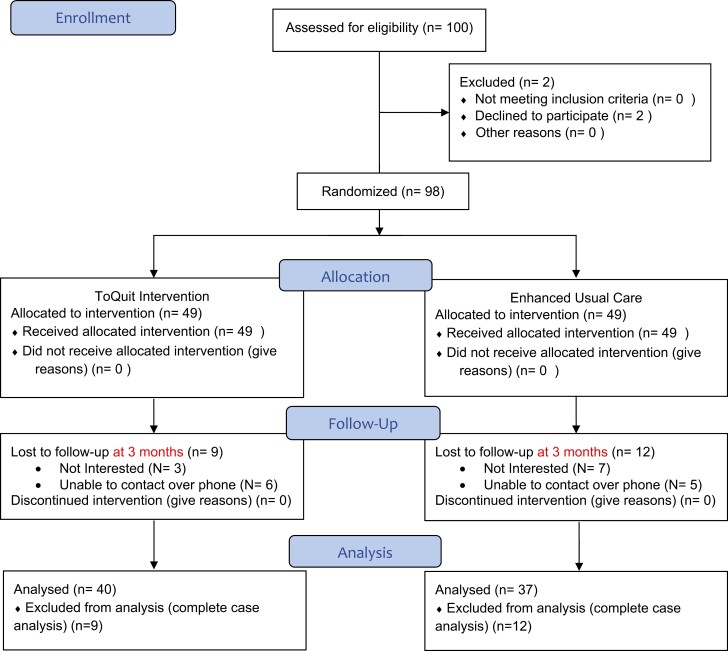
ToQuit pilot randomized control trial CONSORT flow diagram.

Figure 1 summarizes the flow of participants through the trial. One hundred and ninety-one individuals sent in the “ToQuit” message indicating an interest in the study. Of these, we were unable to screen or did not screen 91 for a range of reasons, the commonest being they did not respond when we called back (33%), said that they were not interested in the study (19%), or that they never sent the “ToQuit” message (17%).

Of the 100 screened and eligible for the study, two refused consent because they were “not interested” in the study. The only data we had for those who refused consent was age and there was no statistically significant difference in age between those who consented and those who did not (30.7 [9.1] vs. 26 [1.4]; *p* = .47). Ninety-eight eligible and consenting participants were randomized between the two arms of the trial. Table 1 describes the overall sample and between the two arms on key sociodemographic variables and ASSIST score.

**Table 1.** Baseline Characteristics of Participants

	Total *N* = 98 *N* (%)	ToQuit *N* = 49 *N* (%)	Control *N* = 49 *N* (%)	t or chi^2^	*p*
Mean age in years (SD)	30.7 (9.1)	29.5 (8.6)	31.9 (9.6)	−1.30	0.20
Male gender	97 (99.0)	49 (100.0)	48 (98.0)	1.01	0.32
Age group				2.44	0.30
18 to 30 years	65 (66.3)	36 (73.5)	29 (59.2)		
31 to 50 years	29 (29.6)	11 (22.5)	18 (36.7)		
51 to 65 years	4 (4.1)	2 (4.1)	2 (4.1)		
Marital status				1.09	0.58
Never married	54 (55.7)	28 (57.1)	26 (54.2)		
Married	39 (40.2)	20 (40.8)	19 (39.6)		
Separated/Divorced/Widower	4 (4.1)	1 (2.0)	3 (6.3)		
Education status				0.08	0.99
Completed primary/secondary	19 (19.4)	9 (18.4)	10 (20.4)		
Completed higher secondary	22 (22.5)	11 (22.5)	11 (22.5)		
Graduate	35 (35.7)	18 (36.7)	17 (34.7)		
Postgraduate	22 (22.5)	11 (22.5)	11 (22.5)		
Employment status				3.49	0.06
Employed	81 (82.7)	37 (75.5)	44 (89.8)		
Unemployed/student/retired	17 (17.4)	12 (24.5)	5 (10.2)		
Type of tobacco				0.00	1.0
Smoked	50 (51.0)	25 (51.0)	25 (51.0)		
Smokeless	48 (49.0)	24 (49.0)	24 (49.0)		
Mean ASSIST score (SD)	25.0 (4.1)	25.3 (3.5)	24.8 (4.6)	0.64	0.53

We compared participants who completed outcome assessments and those who did not. 21 (21.4%) participants dropped out from outcome assessments. There were no statistically significant differences between these two types of participants on sociodemographic or tobacco use variables. There was no statistically significant differential dropout by arm (18.4% vs. 24.5%; *p* = .55) or type of tobacco used (16.7% smokeless vs. 26% smoked; *p* = .26). The commonest reasons for dropout were “not interested” (33.3%) and “not responding to follow up phone calls” (42.9%).

There were no statistically significant differences between the two arms at 3 months on any outcomes for the primary (Table 2), secondary, and sensitivity analyses (Appendix S3). For the 77 participants for whom ASSIST tobacco scores were available at baseline and outcome evaluation, there was a significant reduction in the mean score (25.23 [SD = 4.03] vs. 22.61 [SD = 7.55]; *p* = .003) at 3 months.

**Table 2.** Primary and Secondary Outcomes at 3 Months

	Total	ToQuit	Control	*p*	Intervention effect^*^
Point prevalence of self-reported abstinence from tobacco in the past 7 days	16 (20.8)	9 (22.5)	7 (18.9)	0.70	AOR 1.23 (95% CI: 0.38 to 3.97)
Point prevalence of self-reported abstinence from tobacco in the past 28 days	14 (18.2)	8 (20.0)	6 (16.2)	0.67	AOR 1.21 (95% CI: 0.35 to 4.14)
Mean change in ASSIST tobacco score	2.62 (7.49)	3.43 (8.32)	1.76 (6.47)	0.33	SMD −1.74 (95% CI: −5.36 to 1.87)

^*^Adjusted for age category and employment status; AOR = adjusted odds ratio; SMD = standardized mean difference.

### Acceptability, Feasibility, and Perceived Impact

We recruited 21 participants for the qualitative study, of which six were abstinent from tobacco for the past 7 days. The participants’ characteristics are summarized in Table 3 and the key themes are summarized in Table 4.

**Table 3.** Characteristics of Participants in the Nested Qualitative Study

	Total sample *N* = 98 *N*%	Qualitative study sample *N* = 21 *N* (%)
Mean age in years (SD)	30.7 (9.1)	29.6 (7.0)
*Age group*		
18 to 30 years	65 (66.3)	16 (76.2)
31 to 50 years	29 (29.6)	5 (23.8)
51 to 65 years	4 (4.1)	0 (0)
*Marital status*		
Never married	54 (55.7)	12 (57.1)
Married	39 (40.2)	8 (38.1)
Separated/divorced/widower	4 (4.1)	1 (4.8)
*Education status*		
Completed primary/secondary	19 (19.4)	6 (28.6)
Completed higher secondary	22 (22.5)	4 (19.1)
Graduate	35 (35.7)	7 (33.3)
Postgraduate	22 (22.5)	4 (19.1)
*Employment status*		
Employed	81 (82.7)	16 (76.2)
Unemployed/Student/Retired	17 (17.4)	5 (23.8)
*Type of tobacco used*		
Smoked	50 (51.0)	9 (42.9)
Smokeless	48 (49.0)	12 (57.1)
*Abstinent from tobacco at follow-up (past 7 days)*		
Yes		6 (28.6)
No		15 (71.4)
*Abstinent from tobacco at follow-up (Past 28 days)*		
Yes		5 (23.8)
No		16 (76.2)

**Table 4.** Key Themes Related to Acceptability, Feasibility, and Perceived Impact

Theme	Supporting quote
Content and delivery	*“*The information that you send is helpful. One should read it at least two to three times. When you read the information two to three times, ultimately there will be some impact on you. It would be like your friends who consume tobacco- stay away from them, do not meet them or meet them less often. Say no to them. This was the message... Maintain distance from friends who consume tobacco or those who smoke cigarettes because then even you will feel like doing it. This message was also very much helpful.” (43 years, Smoked tobacco, non-abstinent) “A person normally wakes up at eight - eight thirty - nine in the morning. He wakes up between seven to eight. So the person who smokes cigarettes, tries to (smoke). At that time, if he receives that message which tells him to do such and such thing and that he will be saved from the problem and cigarette. Then he will notice more differences.” (43 years, Smoked tobacco, non-abstinent) “There was no difficulty as such. Whenever I would see the message, I would leave my work and read the message first. To see if I get any other idea, or I would check if you tell me anything new to quit it (tobacco).” (27 years, Smokeless tobacco, non-abstinent)
Perceived impact of ToQuit	“Earlier I did not know that all these things happen because of chewing tobacco. Your messages told me about it. Normally many people talk about it even on social media. But they do not explain so much like you did in your message. I have learned that we should have confidence in ourselves. That it is harmful to our body and it reduces our lifespan.” (27 years, Smokeless tobacco, non-abstinent) “Since the time I quit tobacco I am feeling good. Overall there were changes like getting up in the morning, doing yoga, exercise and all meaning the habits which were there one year back, now those habits started again. Improvements such as I used to stay awake till late, was sleeping late so I used to even get up late. Now there is the right routine, the good routine which is there, getting up in the morning at 5 o’clock, exercising, running, walking, so it is a good system.” (23 years, Smokeless tobacco, abstinent)
Barriers	*“*Sometimes, because of work, I wouldn’t be able to read the messages. And second thing is that there are other messages also, there are many other messages that keep coming in. So, you do not understand which one is this message (ToQuit message) when there are so many messages that come in.” *(23 years, Smokeless tobacco, non-abstinent*) “At times there is tension, there is loneliness. So then I have been using it (tobacco) for the past many years since 2012. So, quitting it is very much difficult. Although we want to quit it. Earlier when I had not joined ToQuit, even at that time I wanted to quit it.” (28 years, Smoked tobacco, non-abstinent)
Suggestions to enhance impact	“I think just the messages will not work. If you upload some motivational videos if you include the experiences of such people here, those people who have gone through this, and those who have quit smoking. If you show their experiences, of when they faced difficulties, how they overcame them, then I think that will be more fruitful instead of simple messages. If you show such a person who was once a heavy smoker, and if by using some techniques he has quit smoking, or what are the methodologies that he has used, if you show all these things, then I think that will create more of an impact.” (43 years, Smoked tobacco, non-abstinent)

(1) Content and delivery.

The participants reported appreciating the messages they received in terms of the language used, their comprehensibility, and relevance to the problem that was being addressed.

They reported that the frequency of messages (3–4 days a week) was appropriate. Some indicated that the time at which they received the messages was suitable while others felt that receiving the messages earlier during the day (before their day starts) would have been more impactful.

(2) Perceived impact of the intervention.

Participants reported an overall satisfaction with, and positive impact of the intervention on their lives, and more specifically with enhancing their knowledge about the adverse impacts of tobacco use and changing their attitudes towards using tobacco.

(3) Barriers.

Participants described practical challenges such as not finding enough time to read the messages, the intervention messages getting lost because of several other messages that people keep receiving, and not receiving some of the intervention messages (although our automated delivery system indicated that the message had been delivered). Besides the intervention-related challenges, participants also described challenges related to the addictive nature of tobacco.

(4) Suggestions to enhance impact.

Some participants felt that text messages by themselves might not be sufficient for everyone and that they should be supplemented by medications for those who needed it and the messages could also be enhanced through the use of multimedia content.

## Discussion

Our feasibility RCT has demonstrated that it is feasible to identify and recruit people who wish to discontinue tobacco use and that it is possible to do so via social media platforms. It also demonstrated that it is feasible to deliver a basic mobile-based text messaging intervention to such individuals, that this is acceptable to the target group, and the direction of effect favored text message support over usual care.

The effectiveness of text messaging interventions for health behavior change,^15–17^ and more specifically for tobacco cessation,^18–20^ is well established. However, the evidence for effectiveness of such interventions in LMICs is sparse,^11^ especially in settings where smokeless tobacco use is more common.

In India, the only published study of a text messaging intervention for tobacco cessation was not designed to test effectiveness, but the results do indicate its potential utility.^21^ This intervention (mCessation) differs from ToQuit in several ways. Firstly, it was not designed through a systematic intervention development process and was merely an adaptation of the content from a publicly available message library. mCessation was substantially longer in duration (6 vs. 2 months) but both interventions involved a similar number of messages. Finally, while we evaluated ToQuit through a pilot RCT, the mCessation intervention was examined through an uncontrolled intervention cohort.

There is strong evidence that a range of interventions, both individually and in combination, are effective in increasing the likelihood of smoking cessation.^22^ Despite these interventions being highly competitive public health options to reduce tobacco-related harms, implementation at scale in India has been challenging for several reasons. Both doctors and patients provide and receive only low levels of cessation assistance, respectively, identification of tobacco use and advice given remains inadequate, cessation-related training for medical trainees remains insufficient, and contextual research continues to be limited.^23–26^ All these lead to limited accessibility, acceptability, and adherence to treatment.

For the change in tobacco-use outcomes in our trial, although the changes are in the right direction (ie, higher abstinence and greater reduction in ASSIST score in the intervention arm), there are no significant differences between the arms. Our findings suggest the potential applicability of text messaging interventions for tobacco cessation in low-resource settings. More importantly, a mobile-based intervention is uniquely positioned to overcome some of the key demand (eg, concerns about confidentiality) and supply-side barriers (eg, lack of training of healthcare professionals) to scaling up tobacco cessation interventions, especially in low-resource settings. Additionally, such an intervention ensures the standardization of intervention content by eliminating the variability in quality that can occur when delivered by humans. This is an especially appealing intervention for a country such as India where there is a high (and growing) prevalence of tobacco use and substantial coverage of telecom services −1.2 billion mobile phone subscriptions and a teledensity of 87%.^27^

As expected for a feasibility trial our study was not powered to test effectiveness. Besides the lack of power to examine effectiveness, our study had other limitations. The tobacco use outcomes were based on self-report data only, which could lead to differential social desirability responses between trial arms. Although we had planned to use salivary cotinine tests to supplement the self-reporting of outcomes, we were unable to do that because of COVID-19 restrictions. Recruitment of the sample was through social media dissemination, and this could result in selection bias. Participants recruited through this strategy could possibly be more health conscious and privileged, and hence not representative of the general population. Finally, for the qualitative study, we only interviewed those who completed the outcome evaluation, and it is possible that those who dropped out from follow-up had a sub-optimal experience of the intervention, which will not be reflected in our findings.

Many times, simple, yet established, technologies such as text messaging are often overlooked in favor of more “technologically advanced” systems that have not been fully tested for their acceptability and feasibility in supporting behavior change.^28^ This is despite the fact that text messaging is ubiquitous, allows for real-time exchange (in contrast to multistep active engagement required for apps), is the most widely adopted and least expensive mobile phone function, allows the delivery of a range of behavior change techniques (eg, reinforcement, goal setting, and feedback), and have substantial effectiveness evidence for health behavior change in a range of conditions including diabetes and obesity.^28^ ToQuit provides yet more preliminary evidence on the potential impact and acceptability of a simple, contextually relevant, and scalable intervention suitable for low-resource settings.

The evidence base for effectiveness of tobacco cessation interventions is predominantly derived from high-income countries and implementation of such interventions at scale is a challenge in low-resource settings. As our intervention is designed to be delivered using basic mobile phone technology it is potentially scalable even in low-resource settings. Additionally, unlike other existing text messaging interventions, our intervention caters to the needs of both people who smoked and those who used smokeless tobacco. Considering our feasibility RCT findings, a definitive trial of ToQuit is indicated and if effective, it could be positioned as one of the first-line responses to tobacco use in India and other similar LMICs.

## Supplementary Material

ntae056_suppl_Supplementary_Material

## Data Availability

The data that support the findings of this study are available from the corresponding author, AN, upon reasonable request.
