# Supplementary material for: Text Message-Based Cessation Intervention for People Who Smoked or Used Smokeless Tobacco in India: A Feasibility Randomized Controlled Trial
Source: Nicotine Tob Res. 2024 Mar 12;26(9):1201–8. doi: 10.1093/ntr/ntae056 (PMC11339165; doi:10.1093/ntr/ntae056)
Supplement: ntae056_suppl_Supplementary_Material [file ntae056_suppl_supplementary_material.docx]

**Supplementary Material**

**Appendix 1: Intervention framework and Behaviour Change Techniques (BCTs)**

| **Phases** | **Weeks** |  | **Theme** | **BCTs (target)** |
| --- | --- | --- | --- | --- |
| 1: Orientation | Week1 | Day1 | PSYCHOEDUCATION ABOUT CONSEQUENCES |  |
|  |  | Day2 |  | Information about health consequences |
|  |  | Day3 |  | Pros and cons (of quitting) |
|  |  | Day4 |  | Information about social  and environmental  consequences Information about emotional consequences |
|  | Week2 | Day1 | GOAL SETTING | Goal setting (outcome) |
|  |  | Day2 |  | Commitment (to quit) |
|  |  | Day 3 |  | Goal setting (behaviour) |
| 2: Skill-building | Week3 | Day1 | BEHAVIOURAL STRATEGIES | Self-monitoring of behaviour |
|  |  | Day2 | Reminder about quit date | Information about antecedents Avoidance/reducing  exposure to cues for the  behaviour Restructuring the physical environment |
|  |  | Day 3 |  | Behaviour substitution Distraction |
|  | Week4 | Day1 | COGNITIVE STRATEGIES | Self-talk |
|  |  | Day2 | Reminder about quit date | Reduce negative emotions |
|  |  | Day 3 |  | Comparative imagining of future outcomes |
|  | Week5 | Day1 | ADDITIONAL SUPPORT | Social support (emotional) |
|  |  | Day2 | Reminder about quit date | Social support (unspecified) |
|  |  | Day 3 |  | Social support (unspecified) |
|  | Week6 | Day1 | HANDLING URGES/CRAVINGS | Information about antecedents Self-monitoring of behaviour |
|  |  | Day2 | Reminder about quit date | Instruction on how to perform the behaviour (handling urges) |
|  |  | Day 3 |  | Problem solving  Action planning |
| 3: Relapse prevention | Week7 | Day1 | RELAPSE PREVENTION | Information |
|  |  | Day2 |  | Action planning |
|  |  | Day 3 |  | Pros and cons  Prompts and cues |
|  | Week8 | Day1 | MAINTENANCE |  |
|  |  | Day 2 |  |  |
|  |  | Day 3 |  | Feedback on behaviour |

# Appendix 2: ToQuit pilot RCT: Interview guide for nested qualitative study

Thank you for agreeing to participate in this interview.

You have been part of this mobile-based intervention for the past 8 weeks. During these 8 weeks, you have received messages on your mobile phone in the form of information, tips and reminders to help you quit tobacco.

Over the course of the intervention, you may have found some of the messages helpful or some messages may not have been helpful to you. You may have liked receiving some messages or disliked some messages or parts of the intervention. All these perspectives are valid and will help us to improve this intervention.

Today we will try to understand your overall experience of this intervention. The purpose of this interview is to get your feedback on the feasibility, acceptability and perceived impact of the intervention content and delivery of messages. Your feedback will help us to improve the delivery and content of this intervention, to share it with a greater number of tobacco users with similar challenges.

Let me start by asking you some questions about your overall experience.

## Overall experience

1. Thank you for agreeing to participate in this intervention.

How was your overall experience with this intervention through mobile messaging?

Probes-

- How comfortable were you receiving this intervention over your mobile phone?
- How comfortable were you reading the messages over your mobile phone during the last 8 weeks?

## Perceived impact

1. During this 2-month intervention, were there changes in your tobacco use?

Probes-

- Tell me about the overall change. How did it change? Why do you think this change occurred?

1. How has the participation in our program affected your tobacco use?

Probes-

- How has the way you think about tobacco use changed?
- Can you tell me about what you have learnt about quitting tobacco?
- Can you tell me how you have used this information in your daily life?

## Content

1. I am now going to ask you some questions about the content of our intervention messages. What do you think about the information provided through the intervention?

Probes-

- How helpful did you find this information?
- Is there any particular type of messages (information, advise and tips to quit, reminders) that you found more helpful/less helpful?
- What additional information would be helpful for you?

1. What are your thoughts on the language or wording of the messages?

Probes-

- How easy/difficult did you find the language of these messages?
- How did the language used in the messages influence your participation?
- What language/ wording would be most ideal?

## Delivery

The messages you received included information, tips and reminders about your tobacco use. I am going to ask you about your experience with these messages.

1. What do you think about the length of the messages you received?

Probes-

- How did this affect your participation?
- What length of messages would be ideal?

1. What do you think about the number of messages in a day?

Probes-

- How did this affect your participation?
- What number of messages would be ideal?

1. What are your views on the frequency of the messages (number of days you received messages in a week)?

Probes-

- How did this affect your participation?
- What frequency of messages would be ideal?

1. What are your views on the time or the days on which you received the messages?

Probes-

- How did this affect your participation?
- What time and days would be ideal?

## Situational factors

During the 8 weeks, your participation may have been influenced by your circumstances. I am going to ask you about these factors.

1. Tell me about any circumstances, such as activities or work that you were involved in or any other factors that **helped** your participation in this intervention.
2. Tell me about any circumstances, such as activities or work that you were involved in or any other factors that **stopped/created** challenges in your participation in this intervention.

## Summarizing

1. Is there anything that we have not discussed so far, that you would like to share with us about your overall experience with this Intervention?
2. How can we improve the intervention?

Thank you very much for your time. Your feedback and suggestions are a valuable contribution to help us to develop this intervention.

**Appendix 3: Secondary and sensitivity analyses**

|  | Intervention effect | | | |
| --- | --- | --- | --- | --- |
|  | Multiple imputation | Best case | Worst case | Mean substitution |
| Point prevalence of self-reported abstinence from tobacco in past 7 days | AOR 1.22 (95% CI 0.41-3.60) | AOR 0.94 (95% CI 0.40-2.18) | AOR 1.37 (95% CI 0.45-4.16) |  |
| Point prevalence of self-reported abstinence from tobacco in past 28 days | AOR 1.26 (95% CI 0.40-3.98) | AOR 0.91 (95% CI 0.39-2.15) | AOR 1.37 (95% CI 0.42-4.44) |  |
| Mean ASSIST score | SMD -1.30 (95% CI -4.93-2.34) |  |  | SMD -1.39 (95% CI -4.12-1.34) |
